# Palmitoyl Acyltransferase, *Zdhhc13,* Facilitates Bone Mass Acquisition by Regulating Postnatal Epiphyseal Development and Endochondral Ossification: A Mouse Model

**DOI:** 10.1371/journal.pone.0092194

**Published:** 2014-03-17

**Authors:** I-Wen Song, Wei-Ru Li, Li-Ying Chen, Li-Fen Shen, Kai-Ming Liu, Jeffrey J. Y. Yen, Yi-Ju Chen, Yu-Ju Chen, Virginia Byers Kraus, Jer-Yuarn Wu, M. T. Michael Lee, Yuan-Tsong Chen

**Affiliations:** 1 Institute of Biomedical Sciences, Academia Sinica, Taipei, Taiwan; 2 Graduate Institute of Life Sciences, National Defense Medical Center, Taipei, Taiwan; 3 Institute of Clinical Medicine, National Yang-Ming University, Taipei, Taiwan; 4 Institute of Chemistry, Academia Sinica, Taipei, Taiwan; 5 Department of Medicine, Division of Rheumatology, Duke University Medical Center, Durham, North Carolina, United States of America; 6 Laboratory for International Alliance on Genomic Research, RIKEN Center for Integrative Medical Sciences, Yokohama, Japan; 7 Graduate Institute of Chinese Medical Science, China Medical University, Taichung, Taiwan; 8 Department of Pediatrics, Duke University Medical Center, Durham, North Carolina, United States of America; University of California Davis, United States of America

## Abstract

ZDHHC13 is a member of DHHC-containing palmitoyl acyltransferases (PATs) family of enzymes. It functions by post-translationally adding 16-carbon palmitate to proteins through a thioester linkage. We have previously shown that mice carrying a recessive *Zdhhc13* nonsense mutation causing a *Zdhcc13* deficiency develop alopecia, amyloidosis and osteoporosis. Our goal was to investigate the pathogenic mechanism of osteoporosis in the context of this mutation in mice. Body size, skeletal structure and trabecular bone were similar in *Zdhhc13* WT and mutant mice at birth. Growth retardation and delayed secondary ossification center formation were first observed at day 10 and at 4 weeks of age, disorganization in growth plate structure and osteoporosis became evident in mutant mice. Serial microCT from 4-20 week-olds revealed that *Zdhhc13* mutant mice had reduced bone mineral density. Through co-immunoprecipitation and acyl-biotin exchange, MT1-MMP was identified as a direct substrate of ZDHHC13. In cells, reduction of MT1-MMP palmitoylation affected its subcellular distribution and was associated with decreased VEGF and osteocalcin expression in chondrocytes and osteoblasts. In *Zdhhc13* mutant mice epiphysis where MT1-MMP was under palmitoylated, VEGF in hypertrophic chondrocytes and osteocalcin at the cartilage-bone interface were reduced based on immunohistochemical analyses. Our results suggest that *Zdhhc13* is a novel regulator of postnatal skeletal development and bone mass acquisition. To our knowledge, these are the first data to suggest that ZDHHC13-mediated MT1-MMP palmitoylation is a key modulator of bone homeostasis. These data may provide novel insights into the role of palmitoylation in the pathogenesis of human osteoporosis.

## Introduction

Palmitoylation is a post-translational lipid modification involving the addition of a 16-carbon palmitate on specific cysteine residues of proteins through a thioester linkage [1]. Palmitoylation is unique for being the only lipid modification that has been shown to be reversible; this confers upon it the capability being a dynamic modulator of physiologic and pathologic conditions. To date, numerous proteins have been reported to be palmitoylated including scaffold proteins, ion channels, signaling molecules, cell adhesion molecules, and receptors. Palmitoylation has been shown to be an important regulator of protein trafficking, protein stability, protein-protein interactions and signal transduction [2]–[4].

A family of proteins with palmitoyl acyltransferase (PAT) activity was recently identified in yeast [5], [6]; these proteins contain aspartate-histidine-histidine-cysteine (DHHC) motifs that mediate the PAT enzymatic activity. There are at least 23 DHHC PATs in the mammalian genome [7]. Current knowledge is limited of the involvement of the DHHC family in disease processes. Although *DHHC2* and *DHHC11* were reported to relate to cancers [8]–[10], most of the evidence concerning *DHHC* gene functions has been gleaned in the context of neurological development [11]. To date, 4 mouse models have been generated: *Zdhhc5* gene-trap mice show a reduction in contextual fear [12]; *Zdhhc8* knockout mice manifest a schizophrenia phenotype [13]; mice with a F233 deletion in *Zdhhc21* show abnormalities of skin homeostasis and hair defects [14]; and as described in our previous report, a nonsense mutation was generated in the Zdhhc13 gene by ENU mutagenesis. This mutation resulted in nonsense mediated mRNA decay of Zdhhc13 mRNA. The *Zdhhc13* deficient mice show the most severe phenotype with amyloidosis, alopecia, and osteoporosis [15]. The detailed pathogenic mechanisms of all these phenotypes still remain unclear.

Our goal in this study was to investigate the pathogenic mechanisms underlying osteoporosis in the *Zdhhc13* deficient mice. We aimed to understand how a palmitoylation enzyme, *Zdhhc13*, can affect bone homeostasis with the hope of providing new insights into the biological functions of palmitoylation and the pathogenic mechanisms of human osteoporosis.

## Materials and Methods

### Mice and genotyping

The *Zdhhc13* mutant mice were generated by ENU mutagenesis as described previously [15]. Genotype was analyzed by sequencing tail genomic DNA. Newborn mice were sacrificed by incubation in CO_2_ for 15–20 minutes. All the animals and protocols (IACUC number: 11-05-187) used in this study were approved by the Institutional Animal Care and Utilization Committee of Academia Sinica.

### Skeletal preparation

Newborn mice were skinned and eviscerated. The remaining skeletons were fixed overnight in 1% acetic acid and 95% ethanol, then stained with Alcian blue 8GX (0.05%) for 72 hours, followed by dehydration in 95% ethanol for 24 hours. The solution was changed to 1% KOH until the bone became visible. The skeletons were stained overnight with Alizarin red (0.005%). Specimens were cleared, dehydrated in 70% ethanol/glycerol (1∶1), and finally stored in 100% glycerol.

### Micro-Computed Tomography (MicroCT)

Tissues were fixed in 4% paraformaldehyde overnight and transferred to 70% alcohol. The microCT scan was performed as described previously [15].

### Pathology and Immunohistochemistry (IHC)

Tissues were fixed in 4% paraformaldehyde and decalcified in 10% EDTA. Paraffin sections were stained with Masson’s trichrome stain for morphological analysis. For IHC, antigens of de-paraffinized sections were retrieved by 0.05% trypsin or hyaluronidase (10 mg/ml) and treated with 3% H_2_O_2_. After blocking with 5% normal goat serum, tissues were incubated with primary antibodies in 4°C, overnight. The following rabbit polyclonal antibodies against mouse were used, anti-VEGF (Abcam, Cambridge, UK), anti-PECAM (Abcam), and anti-Osteocalcin (Millipore, Billerica, MA, USA). Sections were then incubated with anti-rabbit secondary antibody (Vectastain ABC system, Vector Laboratories, Servion, Switzerland) and developed with 0.1% 3, 39-diaminobenzidine. Images were captured using standard light microscopy (Zeiss, Oberkochen, Germany) and quantified using Image-Pro Plus software (Rockville, MD, USA). Data from three independent mice staining were used for statistical analysis.

### *In situ* hybridization

RNA *in situ* hybridizations were performed on paraffin sections as previously described [16]. The PCR product (501 bp) generated with primers specific to mouse *Zdhhc13* (Forward: GGGCCATCCGACAAGGGCAT, and Reverse: TGTGCAGCCATCGCCAAAGC) was inserted into pGEM-T Easy vector (Promega, Madison, WI, USA). Digoxigenin (DIG)-labeled single-strand sense and anti-sense RNA probes were prepared with a DIG-RNA labeling kit (Roche, Indianapolis, IN, USA). The hybridized probe-RNA was then detected by anti-DIG-AP (alkaline phosphatase) (Roche) and visualized by NBT/BCIP (Roche) with a developing time of 3 hours.

### RNA isolation and real-time PCR (Q-PCR)

Total RNA was extracted from tissue using the RNeasy kit (Qiagen, Hilden, Germany). cDNA was synthesized from 2 μg total RNA using the SuperScript III First-Strand cDNA Synthesis Kit (Invitrogen, Grand Island, NY, USA) and 15 ng of the cDNA was amplified in a final volume of 15 μl for Q-PCR. Q-PCR was performed using Power SYBER green PCR Master Mix with ABI PRISM 7700 Sequence Detection System (Applied Biosystems, Grand Island, NY, USA). The level of gene expression was normalized to *Gapdh* for the fold change calculation. Primers: *Zdhhc13* F- CAGCAGCATCCATCTGGCGGT; R- GCCGATAGCATGAGCGGCGT; *Gapdh* F-TGCCAAGGCTGTGGGCAAGG; R-TCTCCAGGCGGCACGTCAGA.

### Cell culture

HEK293 and MC3T3E1 cells were purchased and cultured as described by the American Tissue Culture Collection (ATCC). ATDC5 chondrocytic cells were purchased from RIKEN and cultured using F12/DMEM medium (Sigma-Aldrich, Louis, MO, USA) with 10% FBS, 1% penicillin and streptomycin. The primary osteoblasts were isolated by modifying of a previous method [17]. Mainly, femur and tibia were collected from WT and mutant mice at age P14. Bone marrow was flushed out with PBS. The bone tissues were then dissected into 1–2 mm^2^ fragments and digested in collagenase type II solution (Worthington, Lakewood, NJ, USA) for 4 hours. After washing with DMEM, the digested bone fragments were placed in culture dishes or chamber slides for following experiments. Primary epiphyseal chondrocytes were isolated as previously described [18] with several modifications. Briefly, P10 mice were scarified, the epiphysis region was dissected, and the connective tissues were removed. The connective tissue free epiphysis was then digested with type II collagenase (Worthington) overnight. Chondrocytes were separated by 70 μm mesh (BD Biosciences, San Jose, CA, USA) and cultured in DMEM containing 10% FCS. No subculture was performed to avoid transformation of the cell phenotype. Cell lysates were generated with RIPA (Millipore).

### Western blotting (WB)

Tissue or cell lysates were separated by SDS-PAGE gel and transferred to PVDF membrane (Millipore). After blocking with 5% milk, the same antibodies used for VEGF and osteocalcin IHC were used for WB detection. Signals were developed by film after incubating with appropriate horseradish peroxidase (HRP) - conjugated secondary antibodies (Millipore). Densitometry quantification was performed using software in BioSpectrum Imaging System (UVP, Upland, CA, USA). Intensity for all selected bands was all normalized to their actin (loading control) intensity then calculated as fold change to vector group. Data from three independent repeat experiments were used for statistical analysis.

### Co-immunoprecipitation (Co-IP)

Mouse Zdhhc13 cDNA was cloned into a pcDNA4/myc-His expression vector (Invitrogen); mouse MT1-MMP was cloned into a pcDNA3.1/V5-His TOPO TA Expression Vector (Invtrogen). These vectors were co-transfected into HEK293 cells using Lipofectamine 2000 (Invitrogen). 24 to 48 hours after transfection, cells were harvested and lysed with lysis buffer (150 mM Nacl, 50 mM Tris-HCl pH 7.4, 5 mM EDTA, 1 mM PMSF, 1X protease inhibitor cocktail (Roche), 1% TritonX-100). Total Zdhhc13 and MT1-MMP proteins were immunoprecipitated using anti-V5 (Invitrogen) or anti-myc (Millipore) antibodies, respectively with protein G sepharose beads (GE Healthcare, Giles, Buckinghamshire, UK). The Co-IP results were analyzed by WB with the antibodies used for IP.

### Palmitoylation assay (Acyl-biotin exchange assay)

WT and mutant mouse Zdhhc13 cDNA were subcloned into the p3XFLAG-CMV14 vector (Sigma-Aldrich). The MT1-MMP-V5-His plasmid was subsequently co-transfected with the WT Zdhhc13 or mutant Zdhhc13 construct into HEK293 cells using Lipofectamine 2000. Cells and tissues were harvested and lysed in lysis buffer containing 50 mM N-ethylmaleimide. Overexpressed MT1-MMP-V5 was purified from 500 μg total cell lysate by immunoprecipitation with anti-V5 antibody (Invitrogen) and protein G sepharose beads (GE Healthcare) in 4°C overnight. Endogenous MT1-MMP was purified from 1 mg epiphysis tissue lysate by immunoprecipitation with anti-MT1-MMP antibody (Abcam) and protein G sepharose beads (GE Healthcare) in 4°C overnight. The antibody-beads purified MT1-MMP was then used for the acyl-biotin exchange assay as described previously [15], [18]. Palmitoylation level was quantified through biotin signal WB using streptavidin-HRP. Palmitoylation level quantification was performed using BioSpectrum Imaging System (UVP, Upland, CA, USA). For cell base analysis, the MT1-MMP palmitoylation intensity (streptavidin intensity) was first normalized to MT1-MMP loading (myc intensity) and then presented as fold change to vector group. For direct tissue *in vivo* analysis, the palmitoylation level was presented as percentage. It was calculated as streptavidin intensity divided by MT1-MMP intensity.

### Immunofluorescence

Cells were fixed with 4% paraformaldehyde in PBS followed by 0.1% Triton X-100 (in PBS) permeabilized for 10 minutes at room temperature. After blocking with 5% normal goat serum for 1 hour, cells were incubated with primary antibodies overnight. Antibodies and their dilutions used: rabbit anti-MT1-MMP (1∶250; Abcam). The secondary antibodies used here were anti-mouse Alexa Fluor 594 or anti-rabbit Alexa Fluor 488 correspondence to species of primary antibody (1∶500; Molecular Probes, Grand Island, NY, USA). Nuclei were stained by DAPI using ProLong Gold Antifade Reagent (Invitrogen). Images were captured by UltraVIEW (PerkinElmer, Waltham, MA, USA). Granularity and nuclear intensity were calculated by MetaMorph software (Molecular Devices, Sunnyvale, CA, USA). Granularity represented the average speckle number per cell. Nuclear intensity was quantified as average intensity of green fluorescence which was overlapped with blue (DAPI) fluorescence. The quantitative and statistic data was calculated from a total 140 primary cultured osteoblasts or 122 primary chondrocytes from three littermate pairs.

### Statistical analysis

Statistical significance was determined by two-tailed Student's t-test. A *P*-value <0.05 was considered statistically significant (*P-value <0.05, **P-value <0.01, ***P-value <0.001)

## Results

### A *Zdhhc13* mutation results in poor postnatal bone mass accumulation and early onset osteoporosis in mice

**Figure 1:**
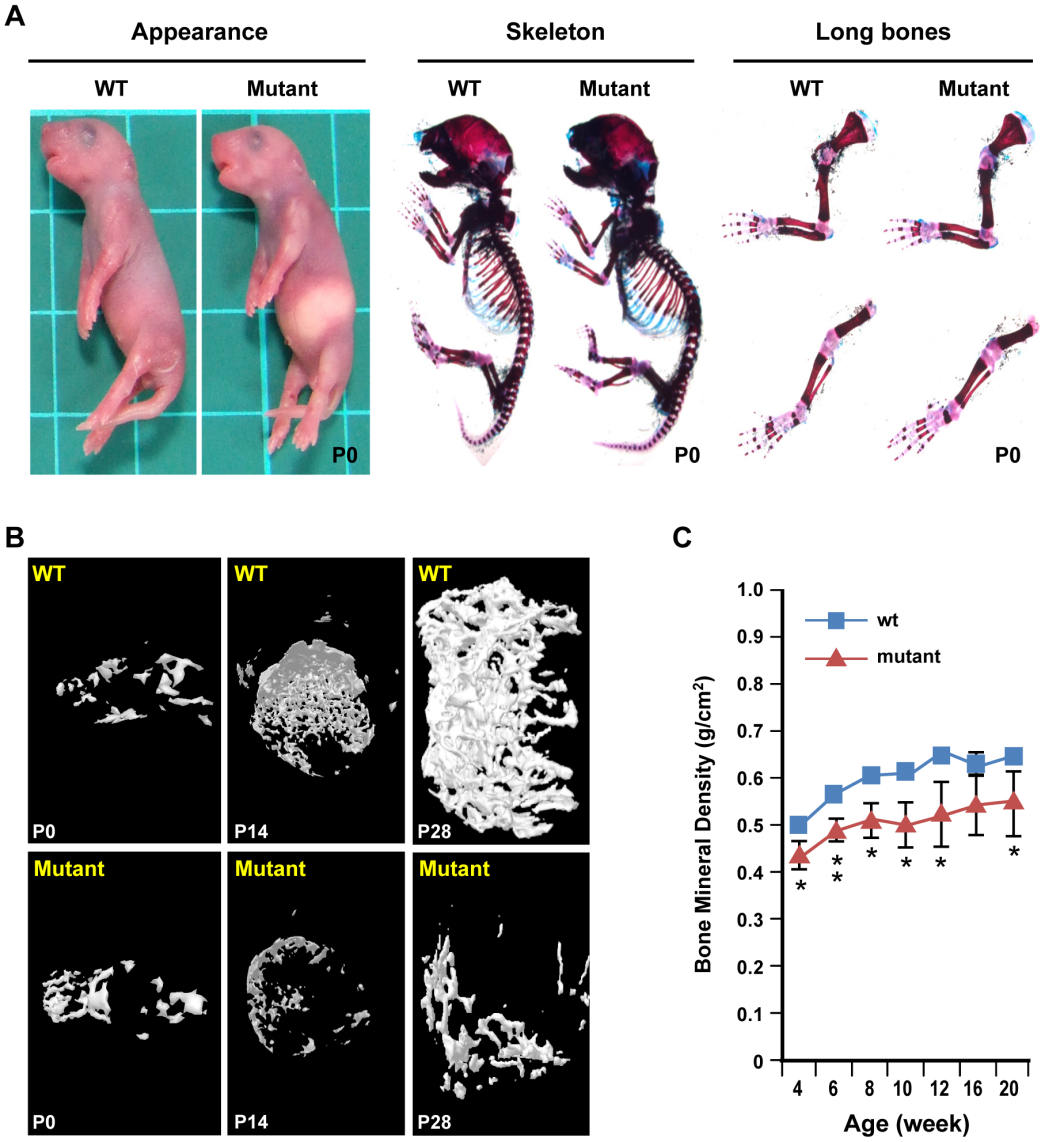
Reduced postnatal skeletal growth and less bone density accumulation in *Zdhhc13* deficient mutation mice. (**A**) WT and mutant mice appearance (left), whole skeleton (middle), and long bone (right) staining with alcian blue (cartilage) and alizarin red S (bone). (**B**) 3D images of trabecular bone constructed μCT scan of P0 (left), P14 (middle), and P28 (right) WT and mutant mice. (**C**) Bone mineral density (BMD) of WT and mutant mice from 4 to 20 weeks of age. Femur BMD was analyzed by μCT. CT scan was performed every 2 weeks starting from 4-week-old to 12-week-old and then every 4 weeks from 12-week-old to 20-week-old. The data were acquired from the same 3 WT and mutant littermate pairs at certain ages. μCT: Microcomputer Tomography. *P<0.05;**P<0.01.

We previously reported that *Zdhhc13* deficient mutant mice had severe osteoporosis at 26 weeks of age [15]. To understand whether the osteoporosis was due to an embryonic or postnatal defect, we first analysed the mice at birth. The appearance of WT and mutant mice was indistinguishable at birth (Figure 1A left). Skeletal staining showed similar bone structure. All the bony components were intact in the mutant mice (Figure 1A middle). The size of long bones was not significantly different between WT and mutant (Figure 1A right). Notable growth differences first appeared on postnatal day 10 (P10) and persisted through the lifespan (P70) (Figure S1). Length of femur showed no significant difference in WT and mutant at P0. At age P14 and P28, the femur was significantly shorter in mutant. (Figure S2). Bone mineral density (BMD) and trabecular bone were similar between WT and mutant mice at birth (Figure 1B left, Table S1). At P14 of age, mutant mice showed significantly lower bone volume, thinner and fewer trabecular bone with larger inter space comparing to WT. The BMD was also significantly lower in mutant (Figure 1B middle, Table S1). By P28, a more severe low bone mass and loosened trabecular bone phenotype was observed in mutant mice. The bone volume and bone density were significantly lower, trabeculaes were thinner and trabecular number was extremely low (Figure 1B right, Table 1). Based on bone mineral density (BMD) by MicroCT, accumulation of bone mass was impaired in mutant mice from 4 to 20 weeks of age (Figure 1C). These results suggest that *Zdhhc13* deficiency impacts postnatal not prenatal bone mass and bone accumulation. Similar bone changes were also observed in *Zdhhc13* gene-trap mice (Figure S3).

**Table 1:** Quantitative μCT results of WT and mutant femur trabecular bone at P28.

	WT	Mutant
**BV/TV (%)**	6.60±2.15	0.89±0.63^**^
**Tb.Th (mm)**	0.07±0.004	0.05±0.004^**^
**Tb.Sp (mm)**	0.39±0.09	0.62±0.04^**^
**Tb.N (1/mm)**	0.99±0.27	0.17±0.17^**^
**SMI**	2.31±0.13	2.68±0.17^**^
**BMD (g/cm^3^)**	0.50±0.01	0.44±0.03^**^

3 WT and mutant mice littermates were analyzed. BV/TV: bone volume/ tissue volume; Tb.Th: trabecular bone thickness; Tb.Sp: trabecular seperation; Tb.N: trabecular bone number; SMI: structure model index; BMD: bone mineral density.

### *Zdhhc13* mutant mice showed delayed epiphyseal maturation, disorganized growth plate structure, and reduced endochondral bone

**Figure 2:**
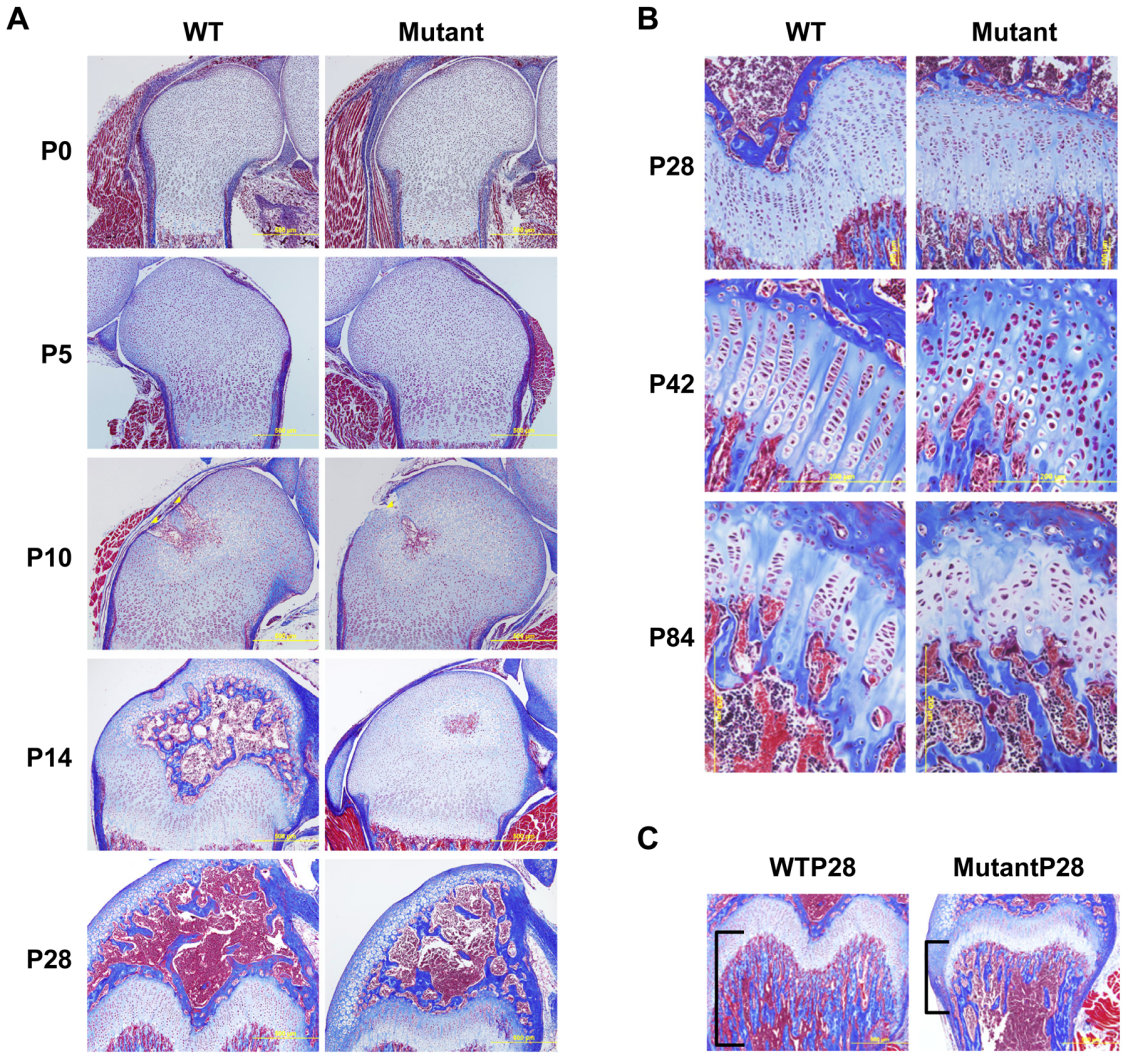
Delayed epiphysis maturation, growth plate disorganization, and diminished endochondral bone in *Zdhc13* deficiency mutation mice. (A) WT and mutant distal femoral epiphysis at age of P0, P5, P10, P14, and P28. Yellow arrows: canals invaded from perichondrium. (B) WT and mutant femoral growth plate at age P28, P42, and P84. (C) Endochondral bone of WT and mutant at P28. Square brackets: the region of trabecular bone. 3 WT and mutant littermate pairs were analyzed by Masson's trichrome stain at each time point.

Histology of the distal femur from birth (P0) to P28 showed a delay in formation of the secondary ossification center (SOC) in mutant mice (Figure 2A). From P0 to P5 the epiphyseal structure was similar between the WT and mutant. Compared to WT, at P10, fewer cartilage canals appeared at epiphyses of mutant mice. By P14, when the SOC cavity was clearly present at WT epiphyses, the SOC cavity remained immature at mutant mouse epiphyses, and was underdeveloped though P28 (Figure 2A bottom). The orientation of the epiphyseal growth plate depends on well-controlled chondrocyte differentiation and proper SOC formation. Even though the SOC was formed at P28 in mutant mice, the well-organized column structure of resting, proliferating and hypertrophic chondrocytes started to be disrupted at P28 (Figure 2B top). Severity of growth plate disorganization increased with age P42 and P84 (Figure 2B). In accord with the microCT data, a significantly reduced endochondral bone was also observed (Figure 2C) suggesting a defect in endochondral ossification. Thus, *Zdhhc13* appears to be crucial for the establishment of appropriate epiphyseal growth plate structure and facilitates endochondral bone formation.

### *Zdhhc13* is detected in a wide range of bone cells with a high level in proliferating and hypertrophic chondrocytes

**Figure 3:**
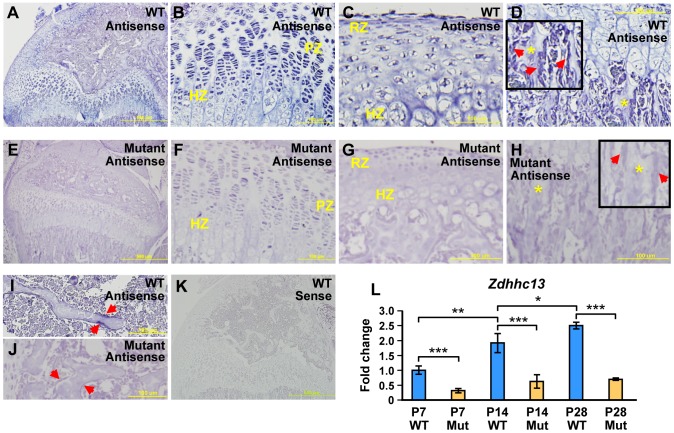
Expression pattern of *Zdhhc13* expression in bone cells. The mRNA expression of Zdhhc13 gene was determined by in situ hybridization using Zdhhc13-specific antisense probes. Expression of *Zdhhc13* in P14 (**A-D, I**) WT distal femur and (**E-F, J**) mutant proximal tibia. Higher power images of (**B**) WT (**F**) mutant hypertrophic and proliferating chondrocytes in the growth plate (**C**) WT (**G**) mutant resting and hypertrophic chondrocytes adjacent to perichondrium (**D**) WT (**H**) mutant osteoblasts (red arrow) surrounding trabecular bone (yellow star) (**I**) WT (**J**) mutant bone lining cells (red arrow). (**K**) Sense probe was used as negative control. (**L**) Postnatal *Zdhhc13* expression pattern in P7, P14, and P28 epiphysis tissue by Q-PCR. 5 WT and mutant littermate pairs were analyzed for each age. HZ: hypertrophic zone; RZ: resting zone; PZ: proliferating zone. Mut: mutant. *P<0.05; **P<0.01; ***P<0.001.

*In situ* hybridization was performed to analyze the expression pattern of *Zdhhc13*. *Zdhhc13* expression signals in P14 WT distal femur (Figure 3A-D and I) and mutant proximal tibia (Figure 3E-H and J) were compared to the negative sense control (Figure 3K). *Zdhhc13* was detected predominantly in proliferating and hypertrophic chondrocytes (Figure 3B). It was also detected in chondrocytes near the perichondrium (Figure 3C) and in osteoblasts surrounding trabecular bone (Figure 3D). Some bone lining cells also showed positive signals (Figure 3I). Since our mutant mice carried a non-sense mutation that led to Zdhhc13 mRNA premature degradation, comparable regions in mutant hypertrophic and proliferating chondrocyte (Figure 3F), perichondrium (Figure 3G), trabecular bone area (Figure 3H), and bone lining cell (Figure 3J) showed very week staining result. We further performed Q-PCR to quantify the *Zdhhc13* level in the WT and mutant mice. Interestingly, Zdhhc13 gene expression increased post-natally and correlated with age after birth in WT (Figure 3L). In contrast, in mutant mice gene expression of *Zdhhc13*, quantified by Q-PCR, was decreased to 40% of WT at P7 and to less than 25% after P14 (Figure 3L).

### ZDHHC13 interacts and palmitoylates MT1-MMP

Previous studies reported a phenotype for MT1-MMP (membrane type 1- matrix metalloproteinase, also known as MMP14)-deficient mice [19], [20] that was remarkably similar to that of the *Zdhhc13* mutant, namely, impaired endochondral ossification, defective angiogenesis, and osteopenia. We hypothesized that MT1-MMP might be a direct palmitoylation substrate of ZDHHC13 and the palmitoylation of MT1-MMP may play a regulatory role in the skeletal system. For this reason we examined the expression and palmitoylation of MT1-MMP in the *Zdhhc13* mutant mice. By immunohistochemistry (IHC) (Figure S4A-F) and Western blot (Figure S4G), WT and mutant Zdhhcc13 epiphyseal tissue had similar levels of MT1-MMP protein expression.

**Figure 4:**
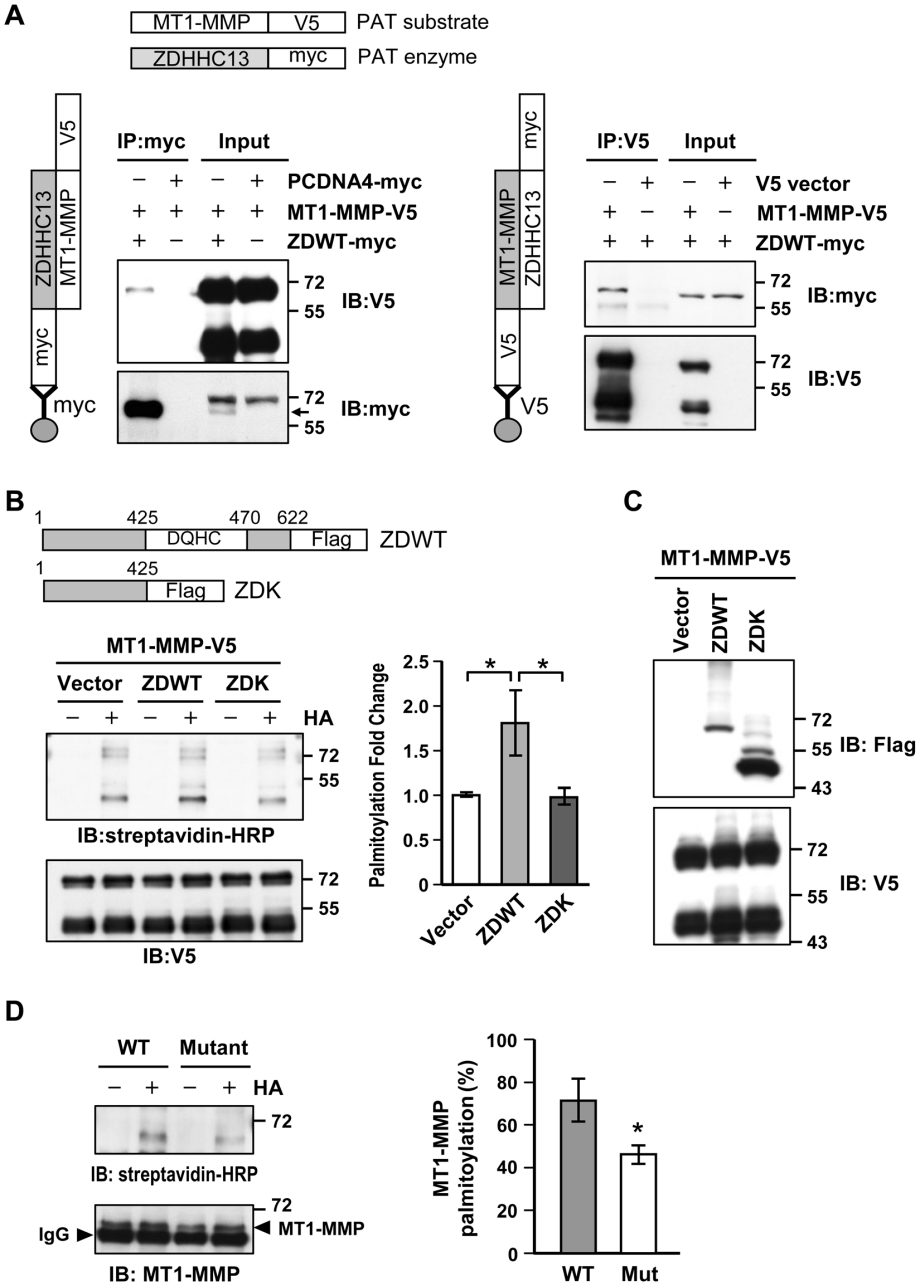
Interaction and direct palmitoylation of MT1-MMP by ZDHHC13. HEK293 cells were co-transfected with ZDHHC13-myc (ZDWT-myc) and MT1-MMP-V5. Co-Immunoprecipitation (IP) were performed to determine interaction between ZDHHC13 and MT1-MMP (**A**) IP using myc antibody (IP: myc) pulled down both ZDWT-myc and MT1-MMP-V5 (left). Arrow indicates the band of ZDWT. Anti-V5 antibody IP (IP: V5) pulled down both MT1-MMP-V5 and ZDWT-myc (right). Vector (PCDNA4-myc) IP group was used as control. Protein lysate before IP was used as input. The cartoon illustrated the constructs and grouping of co-IP experiments. PAT: palmitoyl acyltransferase. (**B**) Palmitoylation level of MT1-MMP examined by acyl-biotin exchange (ABE). ZDHHC13-flag (ZDWT) or ZDK-flag (mimic mutation in our mice) were co-transfected with MT1-MMP-V5 in HEK293 cells. Streptavisdin-HRP signal represented palmitoylation level. Purified MT1-MMP was analyzed by V5 antibody and used as input normalization control. The quantitative palmitoylation level from three independent repeats was shown as fold change to vector (right). MT1-MMP-V5 has multiple molecular weights of ∼66 KDa and ∼45 KDa (self-processed form). The cartoon illustrated the construct of ZDWT and ZDK. Number represented the amino acid position. DQHC is the domain with PAT activity. (**C**) WB examination of ZDWT, ZDK (enzyme), and MT1-MMP-V5 (substrate) expression using flag and V5 antibody respectively. (**D**) Palmitoylation of MT1-MMP in WT and mutant distal femur epiphysis. MT1-MMP was directly purified from P14 WT and mutant epiphysis protein lysate to perform ABE. Streptavidin-HRP signal represented palmitoylation level. Purified MT1-MMP was analyzed by specific antibody. Quantitative result from three independent experiments of three P14 littermate pairs was shown as palmitoylation percentage (right). *P<0.05.

Finally, co-immunoprecipitation (Co-IP) was performed using ZDHHC13-myc (ZDWT-myc) and MT1-MMP-V5 co-overexpressing HEK293 cells to examine whether ZDHHC13 interacted with MT1-MMP. MT1-MMP-V5 was successfully immunoprecipitated with anti-myc antibodies (Figure 4A left) and conversely, ZDHHC13-myc was successfully immunoprecipitated by anti-V5 antibodies (Figure 4A right). In support of the specificity of the interaction between ZDHHC13 and MT1-MMP, the vector immunoprecipitation controls had no signal.

We used acyl-biotin exchange (ABE) to evaluate the palmitoylation level of MT1-MMP. A ZDHHC13-mutant-Flag (ZDK) expression vector, without the DHHC domain, was constructed to mimic the *Zdhhc13* mutation in our mouse model. MT1-MMP-V5 was co-overexpressed with Flag vector, ZDHHC13-Flag-WT (ZDWT) or ZDHHC13-mutant-Flag (ZDK) in HEK293 cells. Results of ZDWT and ZDK were compared to Flag vector control which indicating basal palmitoylation level. The palmitoylation level of MT1-MMP-V5 was quantified to be nearly 2 fold higher in the ZDWT co-expresssing group than in the vector control or ZDK (mutant mimicking construct) co-expressing groups (Figure 4B). Protein expression of transfected MT1-MMP was comparable with the exception of ZDWT for which it was slightly less (Figure 4C). Abolishment of the palmitoylation signal by palmitoylation inhibitor, 2-bromopalmitate (2-BP), evidenced the MT1-MMP palmitoylation and the cysteine 574 of MT1-MMP was confirmed to be the palmitoylation site by mutagenesis (Figure S5). Finally, we confirmed the ability of Zdhhc13 to palmitoylate MT1-MMP *in vivo* using ABE with direct immunoprecipitation of MT1-MMP protein from P14 epiphyses of WT and mutant mice. MT1-MMP in WT mice was 70% palmitoylated compared with only 40% in mutant mice (Figure 4D). These results confirm that ZDHHC13 is a PAT responsible for a substantial amount (30%) of MT1-MMP palmitoylation.

### Palmitoylation affects the subcellular distribution of MT1-MMP

**Figure 5:**
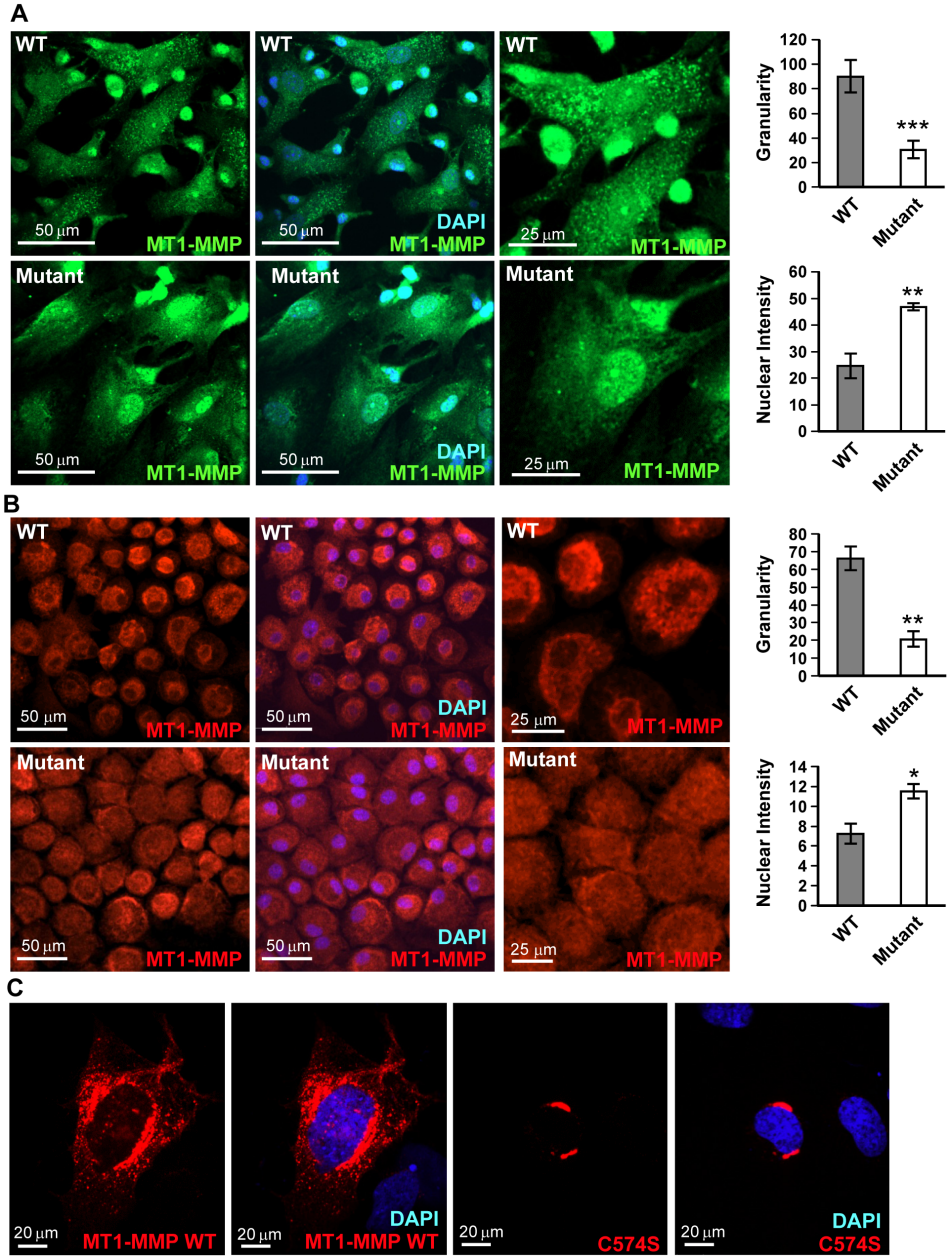
Effect of palmitoylation on MT1-MMP subcellular distribution. (**A**) Immunofluorescence (IF) microscopy of primary osteoblasts from P14 WT (upper panel) and mutant (lower panel) long bone showing MT1-MMP subcellular localization. The granularity (average speckle number per cell) and nuclear intensity quantification results were shown (right). MT1-MMP: Alexa Fluor 488 (green); nucleus: DAPI (blue). (**B**) IF microscopy of primary chondrocytes from P10 WT (upper panel) and mutant (lower panel) epiphysis showing MT1-MMP cellular localization. The granularity (average speckle number per cell) and nuclear intensity quantification results were shown (right). MT1-MMP: Alexa Fluor 594 (red); nucleus: DAPI (blue). (**C**) IF microscopy of ATDC5 cells transfected with WT MT1-MMP-V5 (upper panel) and C574S MT1-MMP-V5 (lower panel). MT1-MMP distribution was analyzed by V5 primary antibody and visualized with Alexa Fluor 594 (red). Nucleus: DAPI (blue). *P<0.05;**P<0.01; ***P<0.001.

As palmitoylation is highly involved in regulating protein subcellular trafficking, we further explored the subcellular localization of MT1-MMP in primary osteoblasts and chondrocytes. While MT1-MMP actively formed cytoplasmic speckles in WT primary osteoblasts, a significantly 60% fewer speckles with 50% increased nuclear localization were observed in mutant primary osteoblasts (Figure 5A). The reduction in MT1-MMP cytoplasmic speckles was also observed and quantified to similar degree in mutant primary epiphyseal chondrocytes when compared to WT (Figure 5B). To further confirm the importance of this palmitoylation on MT1-MMP distribution, MT1-MMP WT and the C574S construct were overexpressed in the chondrocytic ATDC5 cells. WT MT1-MMT formed a cytoplasmic speckled pattern but C574S showed a more condensed pattern in the perinuclear region (Figure 5C). Palmitoylation was known to affect clathrin-mediated MT1-MMP trafficking [21]. We observed 15% reduction of MT1-MMP-clathrin co-localization in *Zdhhc13* mutant primary osteoblasts (Figure S6). These results suggested that cysteine 574 palmitoylation was important for the subcellular localization of MT1-MMP.

### MT1-MMP palmitoylation is associated with VEGF expression in chondrocytes and osteocalcin level in osteoblasts

**Figure 6:**
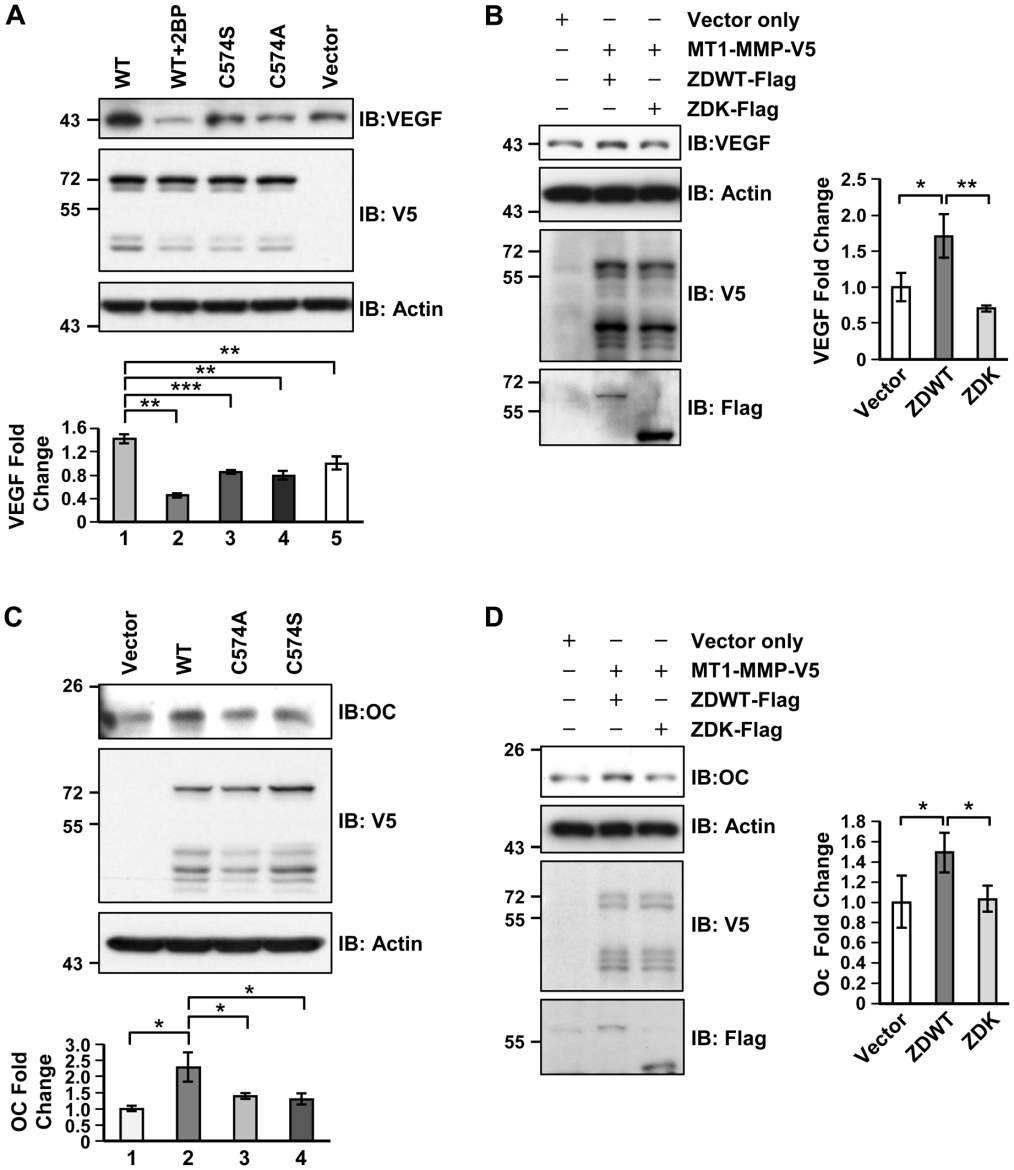
ZDHHC13- mediated MT1-MMP palmitoylation is associated with VEGF expression in chondrocytes and osteocalcin expression in osteoblast. (**A**) WB of VEGF in ATDC5 cells transfected with WT MT1-MMP or blocking it’s palmitoylation by either 2-BP treatment or cysteine 574 mutagenesis (C574A/S). Quantitative fold change to vector was shown below the blot. The number represents lane number from left to right. (**B**) WB of VEGF in ATDC5 cells co-overexpressing WT ZDHHC13 (ZDWT) or mutant ZDHHC13 (ZDK) with WT MT1-MMP. Quantitative fold change to vector was shown (right). (**C**) Osteocalcin (OC) level in MC3T3E1 cells overexpressed with WT MT1-MMP and blocking MT1-MMP palmitoylation by mutant construct (C574A/S). Quantitative fold change to vector was shown below the blot. The number represents lane number from left to right. (**D**) OC level in MC3T3E1 cells co-overexpressing WT ZDHHC13 (ZDWT) or mutant ZDHHC13 (ZDK) with WT MT1-MMP. Quantitative fold change to vector was shown (right). 2BP: 2-bromopalmitate. *P<0.05;**P<0.01; ***P<0.001.

To determine the effects of ZDHHC13-mediated MT1-MMP palmitoylation in the skeletal system, mutagenesis was performed to disrupt cysteine 574 (C574) palmitoylation. The C574 was converted to alanine (MT1-MMP C574A-V5) or serine (MT1-MMP C574S-V5). MT1-MMP WT, C574A, C574S and V5 vector control were transfected into a mouse chondrocyte cell line, ATDC5. VEGF expression was analysed using Western blot. VEGF level was significantly elevated 1.5 fold in the WT MT1-MMP overexpressing group comparing to vector control. The elevation was abolished in C574A and C574S overexpressing groups. Palmitoylation of MT1-MMP WT was inhibited using 4-hour 25 μM of 2-BP treatment. 2B-P inhibition of MT1-MMP WT palmitoylation resulted in greater inhibition of VEGF expression (Figure 6A). To mimic the condition in our mutant mice and confirm the involvement of ZDHHC13, ATDC5 cells were co-transfected with ZDWT and MT1-MMP WT or ZDK and MT1-MMP WT. Western blot showed that, with comparable MT1-MMP expression, the ZDWT but not ZDK group was able to elevate VEGF level (Figure 6B). In addition to chondrocytes, we also explored the effects of MT1-MMP palmitoylation in osteoblasts. The mouse osteoblastic cell line, MC3T3E1, was transfected with MT1-MMP WT, C574A, or C574S construct. The maturation and ossification marker osteocalcin was analysed by Western blot. The expression of osteocalcin increased 2 fold when overexpressing WT MT1-MMP. The incensement was significantly attenuated by C574 mutation (Figure 6C). Likewise, MC3T3E1 cells were co-transfected with ZDWT and MT1-MMP WT or ZDK and MT1-MMP WT to mimic our mouse model. Western blot demonstrated that, under similar MT1MMP expression level, ZDWT but not ZDK expression was able to significantly increase osteocalcin level (Figure 6D). Collectively, these results indicated that ZDHHC13-mediated MT1-MMP palmitoylation positively associated with VEGF expression in chondrocytes and osteocalcin expression in osteoblasts.

### Reduced VEGF level associated with less vascularity in *Zdhhc13* mutant epiphysis and the reduced osteocalcin expression at hypertrophic cartilage and bone surface in *Zdhhc13* mutant mice

**Figure 7:**
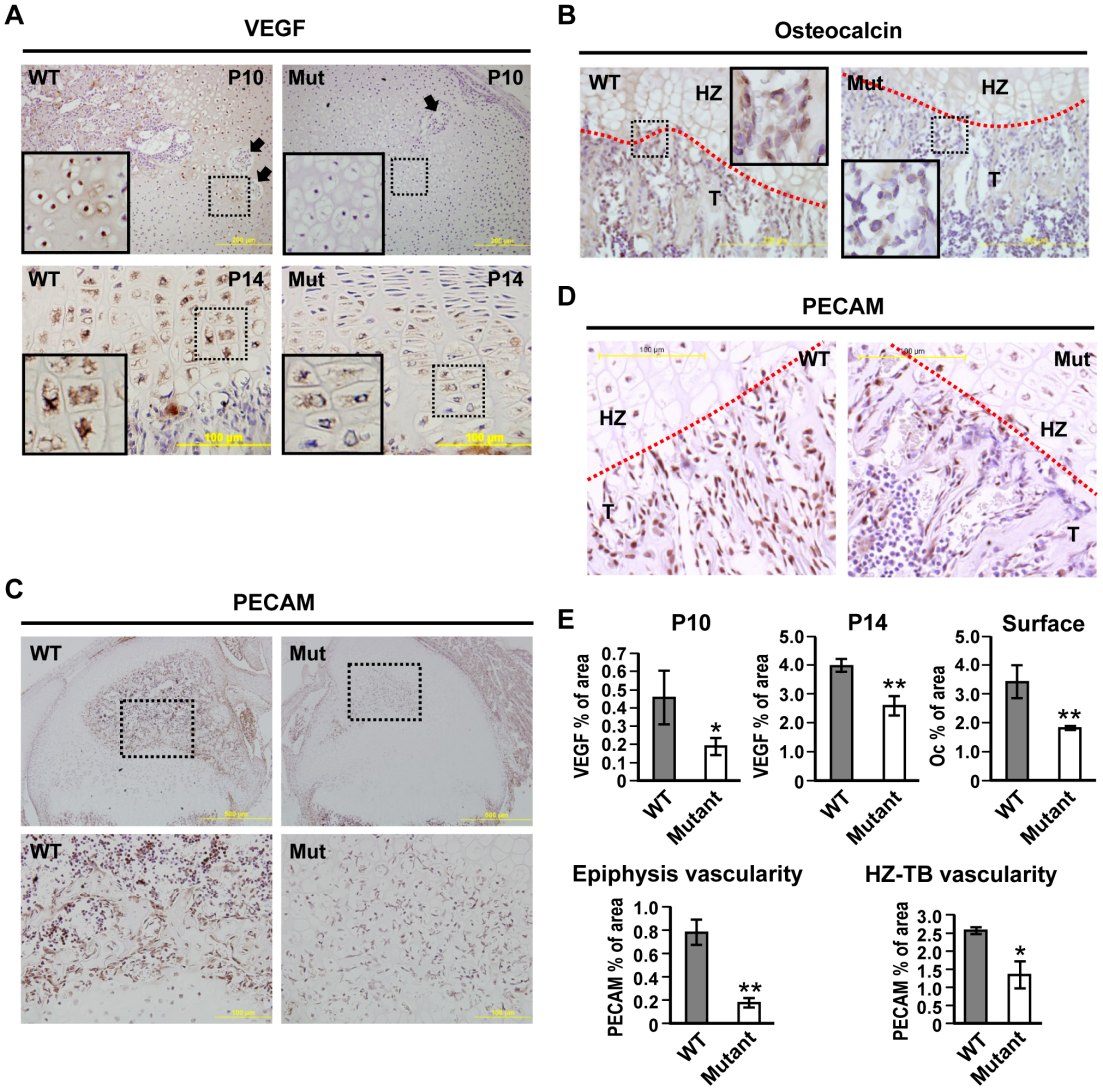
Reduced VEGF and osteocalcin expression with reduced vascularity in *Zdhhc13* deficiency mutation mice. Immunohistochemistry (IHC) of (**A**) VEGF in P10 (upper panel) and P14 (lower panel) WT and mutant distal femoral epiphysis. Black arrows indicate the cartilage canals surrounded by hypertrophic chondrocytes. Dashed-squares indicated the emphasized area shown in bottom left. (**B**) Osteocalcin in P14 WT and mutant epiphysis growth plate. Dashed-squares indicate the emphasized area. (**C**) Endothelial marker PECAM in P14 epiphysis SOC (upper panel) and higher magnification of dashed-area showing PECAM positive cells aligned cartilage canals and SOC cavity (lower panel). (**D**) PECAM in P14 WT and mutant epiphysis growth plate. (**E**) Quantitative results of VEGF, Oc and PECAM IHC. The results were demonstrated as % of area. VEGF staining was quantified as positive stained area among HZ region (represents panel A). Oc staining was quantified as positive stained area on trabecular bone surface (represents panel B). PECAM quantification was separated in to positive stained area within epiphysis (epiphysis vascularity, represents panel C) and positive area among HZ and trabecular bone (HZ-TB vascularity, represents panel D). The statistic comparison was performed using three WT and three mutants IHC in each group. Mut: mutant; HZ: hypertrophic zone; T: trabecular bone; red dash lines: interface of cartilage and bone. *P<0.05;**P<0.01.

To evaluate the effects of ZDHHC13- mediated MT1-MMP palmitoylation on VEGF and osteocalcin *in vivo*, VEGF and osteocalcin IHC was performed on WT and mutant epiphyses. Reduced VEGF level was observed in mutant hypertrophic chondrocytes near cartilage canals (Figure 7A upper panel) and in growth plate (Figure 7A lower panel). Reduced osteocalcin expression was also detected at the hypertrophic zone cartilage (calcified region) and cells on trabecular bone surface of the mutant (Figure 7B). We finally examined whether the reduced VEGF expression was associated with changes in the vascularity of the epiphysis. Staining with the endothelial marker PECAM was performed. Significantly fewer PECAM positive cells were shown in mutant SOC (Figure 7C) and hypertrophic zone- trabecular bone region (Figure 7D). The IHC quantification results (Figure 7E) demonstrated that mutant VEGF level decreased to 50% and 60% of WT in P10 and P14, respectively. The osteocalcin also reduced significantly to half of WT in mutant bone surface area. The vascularized area of mutant epiphysis and hypertrophic- trabeculae region were only 25% and 50% of WT, respectively. These results suggested that ZDHHC13- mediated MT1-MMP palmitoylation is a candidate to cause delayed SOC formation and reduced endochondral bone formation through regulating VEGF and osteocalcin expression in the skeletal system.

## Discussion

Osteoporosis is the most common metabolic bone disorder in elders [22]. Bone density acquired during childhood development and early adult age impacts the incidence of osteoporosis in later life [23], [24]. Recent genome-wide association studies have shown that osteoporosis and BMD are associated with genes participating in skeletal development, bone cell differentiation, and endochondral ossification [25]. In this study we newly identified a molecule, Zdhhc13, as critical for endochondral bone synthesis and normal bone structure.

In the present study, using histology and microCT, *Zdhhc13* mutant mice clearly demonstrated a delay in SOC formation with disorganized growth plate structure, short long bone and diminished endochondral bone formation with poor postnatal bone mass accumulation and a bone phenotype compatible with severe osteoporosis. While our experiments were carried out in the ENU-generated *Zdhhc13* nonsense mutation mouse model which had reduced expression of *Zdhhc13*, we have also validated the osteoporosis phenotype in our gene-trap mice (Figure S3). Although a recent study of the *Zdhhc13* gene-trap mouse model reported a Huntington’s disease phenotype [26], we did not observe this phenotype in our *Zdhcc13* deficient mouse model.

To investigate the possible function of *Zdhhc13* in the skeletal system, we explored the expression of *Zdhhc13* in bone cells. We determined that Zdhhc13 mRNA was detected in a variety of cells, importantly, in osteoblasts and at especially high levels in proliferating and hypertrophic chondrocytes. The expression level of Zdhhc13 mRNA increased from the first week after birth in WT but was dramatically degraded in mutant due to the nonsense mutation (Figure 3). These data suggested the critical function of *Zdhhc13* in postnatal bone growth.

Since ZDHHC13 is a PAT, we speculated that its substrates might mediate regulation of bone growth and development. MT1-MMP is an important factor that governs skeletal development. A previous study reported that MT1-MMP is palmitoylated at cysteine 574 (C574) [21]. Further, MT1-MMP-deficient mice had defects in SOC maturation and endochondral bone formation, as well as kyphosis, osteopenia, dwarfism, and short lifespan [19], [20]. Although, our Zdhhc13 mutant mice were not MT1-MMP deficient, we demonstrated for the fist time that MT1-MMP is a direct substrate of ZDHHC13. The palmitoylation was evidenced by 2-BP treatment and MT1-MMP C574 mutation. MT1-MMP was confirmed to be under palmitoylated in mutant mice (Figure 4). Clathrin-mediated MT1-MMP endocytosis has been shown to be regulated by palmitoylation of MT1-MMP [32]. However, the *Zdhhc13* deficiency mutation had only a minor effect (∼15% reduction) on clathrin-mediated MT1-MMP endocytosis in osteoblasts (Figure S6). We did however find an altered subcellular distribution of MT1-MMP based on palmitoylated state - less cytoplasmic speckle with more nuclear localization- in the *Zdhhc13* mutant in both primary osteoblasts and chondrocytes. Mutation of MT1-MMP C574 also certified our finding of palmitoylation on MT1-MMP subcellular distribution. Since protein interactions can be regulated by palmitoylation [27], we speculated that the altered subcellular speckle localization of under palmitoylated MT1-MMP adversely impacted key regulatory bone functions by MT1-MMP.

MT1-MMP regulates the skeletal system in diverse ways. Manduca, et al. reported the critical temporal regulation of MT1-MMP in governing osteogenesis and mineralization in osteoblasts [28]. MT1-MMP was also shown to be an essential factor in osteocytogenesis through its proteolytic activity [29]. The ability to shed RANKL and ADAM9 makes it both a negative regulator in local osteoclastogenesis [30] and a positive modulator in calvarial osteogenesis [31]. Tang et al. reported recently that MT1-MMP- dependent extracellular matrix remodelling is able to mediate integrin signalling and determine skeletal stem cell differentiation [32]. Independent of its catalytic activity, the cytoplasmic tail (where the palmitoylation site locates) of MT1-MMP is required for myeloid cell nuclear fusion to form osteoclasts [33]. The cytoplasmic tail of MT1-MMP is also able to induce VEGF expression in cancer cells [34]. VEGF is one of the essential factors in bone formation [35]. For long bones to grow, endochondral ossification consistently deposits new bone by replacing cartilage. Cartilage is unique in its avascular nature. Thus, penetration of a well-functioning vascular system is the foundation for the establishment of the growth plate and endochondral ossification [36]. Mice lacking VEGF showed impaired SOC maturation and endochondral ossification due to lack of vascularity [35], [37]. Here we demonstrated that ZDHHC13-mediated MT1-MMP palmitoylation was associated with facilitating VEGF expression in *in vitro* chondrocytic ATDC5 cell system. The reduction of VEGF was also observed and quantified to be 50%- 60% of WT in *Zdhhc13* mutant hypertrophic chondrocytes. A 75% less vascularity was further shown by PECAM IHC in mutant epiphysis. The vascularity also revealed to be only 50% of WT in HZ-TB region of *Zdhhc13* mutant mice. Besides, osteocalcin expression was found to be associated with ZDHHC13-mediated MT1-MMP palmitoylation in MC3T3 cells and in cartilage-bone interface. Collectively, these suggested that *Zdhhc13*-mediated MT1-MMP palmitoylation was a novel regulator in skeletal vascularity and endochondral ossification. These also highlighted the potential diverse roles of ZDHHC13-mediated MT1-MMP palmitoylation in modulating bone homeostasis.

A PAT enzyme, Zdhhc13, may have numerous downstream substrates. A matrix metalloproteinase, MT1-MMP, may also have diverse substrates or interacting molecules. In this complex regulatory network we revealed a novel potential pathway involving regulation of bone formation, development and structure by palmitoylation. The involvement of other ZDHHC13 palmitoylation targets or MT1-MMP substrates is worthy of further investigation.

## Conclusions

To the best of our knowledge, this is the first report showing involvement of a DHHC PAT enzyme, *Zdhhc13*, in epiphyseal maturation and endochondral bone formation. This study revealed a novel pathogenic mechanism of osteoporosis governed by palmitoylation.

## Supporting Information

Figure S1 **Postnatal growth retardation of** ***Zdhhc13*** **deficient mutation mice.** Appearance of WT and mutant mice at age P10, P14, P28, and P70.
(TIF)

Figure S2 **Bone length of P0, P14 and P28** ***Zdhhc13*** **WT and mutant mice.** Femurs were dissected and their lengths were measured. The values from 3 WT and 3 mutant were shown as mean±SD. The square is in 1 mm×1 mm of size. Statistical significance was determined by two-tailed Student's t-test. A *P*-value <0.05 was considered statistically significant (*P-value <0.05, **P-value <0.01). The yellow color of P0 femurs was resulted from fixation in Bouin's solution.
(TIF)

Figure S3 **Bone phenotype in** ***Zdhhc13*** **gene-trap mice.** MicroCT **(A)** 3D images and **(B)** bone volume/ tissue volume (BV/TV) ratio of trabecular bone in *Zdhhc13* WT and gene-trap (Gt) (5 month of age). The method for generation of this gene-trap model was described in our previous paper [15].
(TIF)

Figure S4 **Comparable MT1-MMP expression in WT and mutant epiphysis and primary chondrocytes.** MT1-MMP IHC of P10 **(A-C)** WT and **(D-F)** mutant distal femoral epiphyseal sections. High power view of **(B)** WT and **(E)** mutant mice MT1-MMP expression in chondrocytes around cartilage canal. MT1-MMP expression in **(C)** WT and **(F)** mutant growth plate chondrocytes. Yellow star indicted the canal that will contribute to future SOC formation. **(G)** Expression of MT1-MMP in P14 epiphysis tissue by WB and quantitative results from three WT and mutant littermate pairs (below).
(TIF)

Figure S5 **Abolishment of palmitoylation of mutant MT1-MMP (C574A, C574S) and 25 μM, 50 μM, 100 μM 2-Bromopalmitate treatment.** Palmitoylation level was examined by acyl-biotin exchange. MT1-MMP-V5 WT or C574A or C574S were overexpressed in HEK293 cells. Palmitate was switched to biotin and detected by streptavidin-HRP. Purified MT1-MMP was analyzed by V5 antibody. Red arrows indicate the palmitoylation signals of WT MT1-MMP which was not detected in C574 mutation constructs and 2-BP treatment groups. The 2-BP concentration of 25 μM was used in further experiment. HA: hydroxylamine; 2BP: 2-bromopalmitate.
(TIF)

Figure S6 **Clathrin-mediated MT1-MMP trafficking in WT and** ***Zdhhc13*** **mutant primary osteoblast. (A)** Localization patterns of MT1-MMP in primary osteoblast (OB) from P14 WT and mutant femur. MT1-MMP staining (green), Clathrin staining (red), and merge of MT1-MMP and Clathrin (yellow shows the colocalization of two proteins) in WT (upper panel) and mutant (lower panel) primary OB. White arrows indicated non-clathrin colocalized speckles. **(B)** Colocalization MT1-MMP and clathrin quantitative data. *P-value <0.05, t-test.
(TIF)

Table S1 **Quantitative microCT results of WT and mutant femur trabecular bone and bone mineral density (BMD) at P10 and P14.** 3 WT and 3 mutants were analyzed at each time point. Data was presented as average ± standard deviation. Statistical significance was determined by two-tailed Student's t-test. A P-value <0.05 was considered statistically significant (*P <0.05, **P <0.01). BV/TV: bone volume/ tissue volume; Tb.Th: trabecular bone thickness; Tb.Sp: trabecular separation; Tb.N: trabecular bone number; SMI: structure model index.
(DOCX)
