# Supplementary figures and images for: Palmitoyl Acyltransferase, Zdhhc13, Facilitates Bone Mass Acquisition by Regulating Postnatal Epiphyseal Development and Endochondral Ossification: A Mouse Model
Source: PLoS One. 2014 Mar 17;9(3):e92194. doi: 10.1371/journal.pone.0092194 (PMC3956893; doi:10.1371/journal.pone.0092194)

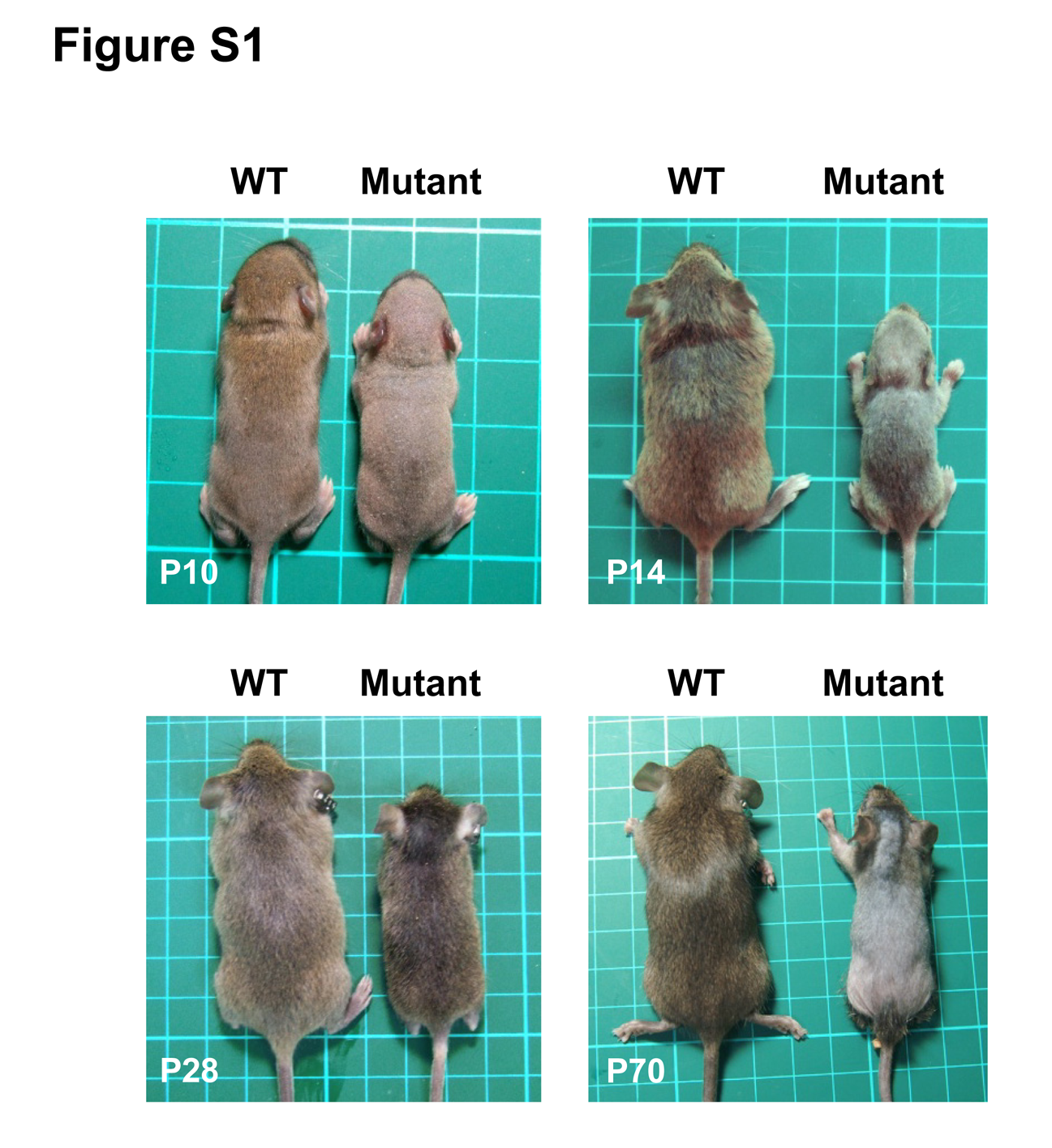

Supplement: Figure S1 — Postnatal growth retardation of Zdhhc13 deficient mutation mice. Appearance of WT and mutant mice at age P10, P14, P28, and P70. (TIF) [file pone.0092194.s001.tif]

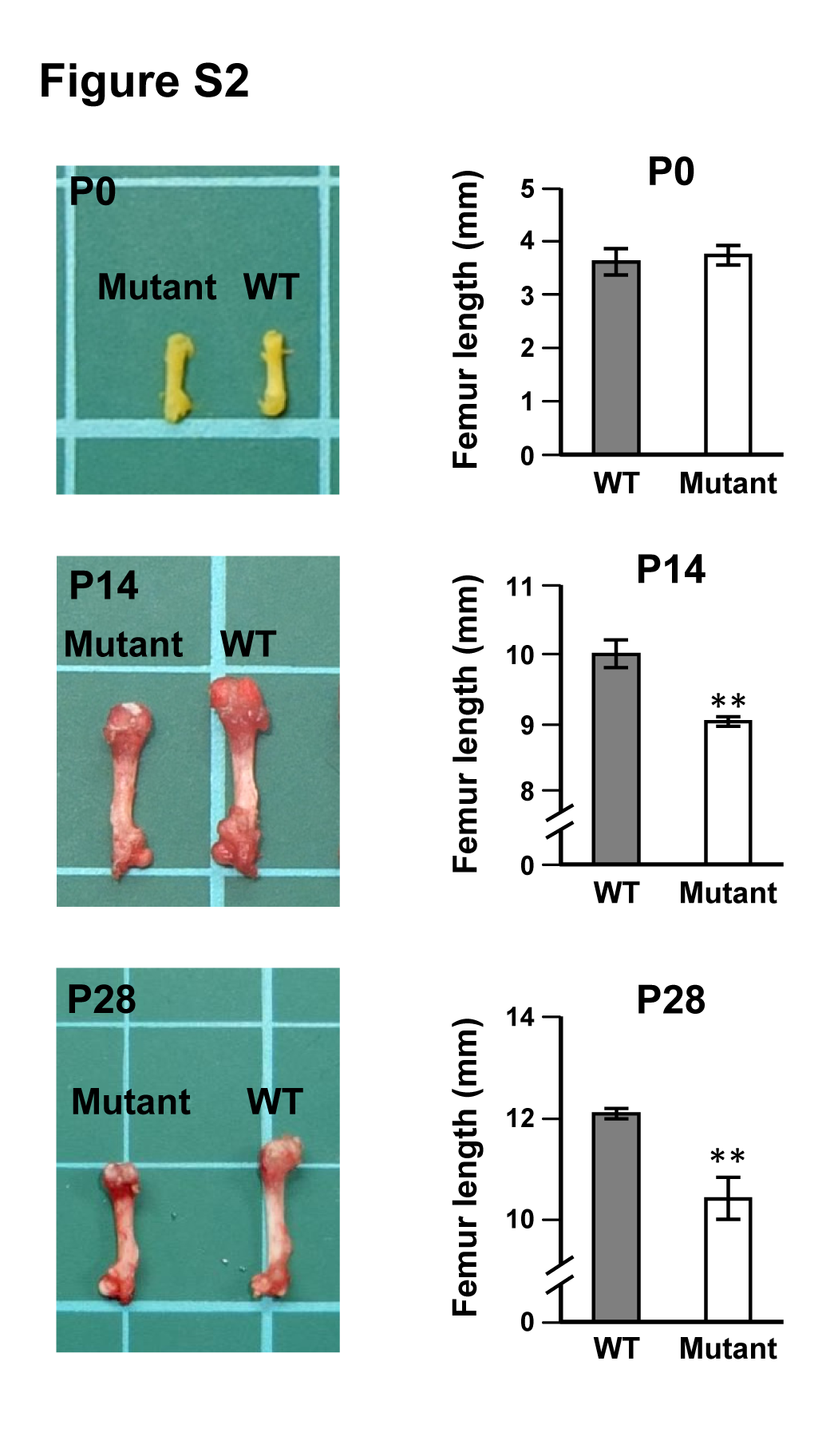

Supplement: Figure S2 — Bone length of P0, P14 and P28 Zdhhc13 WT and mutant mice. Femurs were dissected and their lengths were measured. The values from 3 WT and 3 mutant were shown as mean±SD. The square is in 1 mm×1 mm of size. Statistical significance was determined by two-tailed Student's t-test. A P-value <0.05 was considered statistically significant (*P-value <0.05, **P-value <0.01). The yellow color of P0 femurs was resulted from fixation in Bouin's solution. (TIF) [file pone.0092194.s002.tif]

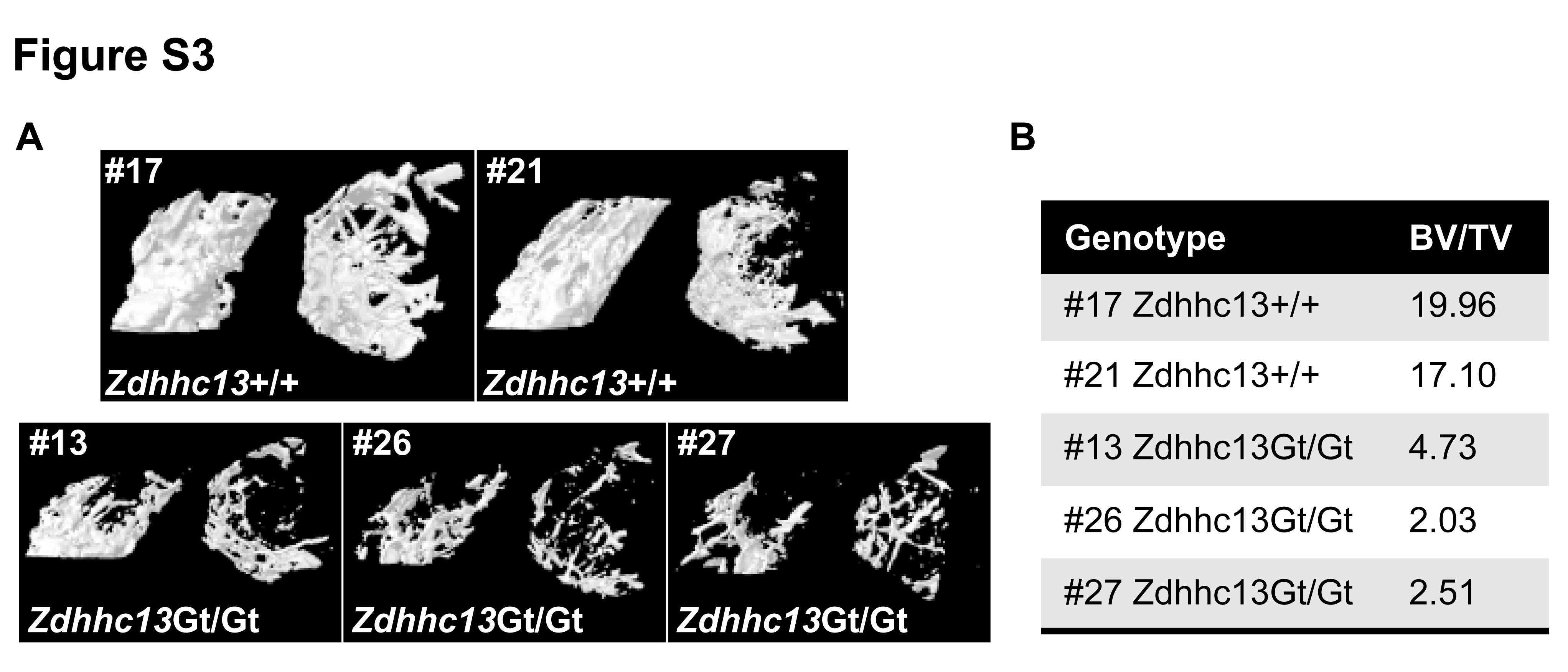

Supplement: Figure S3 — Bone phenotype in Zdhhc13 gene-trap mice. MicroCT (A) 3D images and (B) bone volume/ tissue volume (BV/TV) ratio of trabecular bone in Zdhhc13 WT and gene-trap (Gt) (5 month of age). The method for generation of this gene-trap model was described in our previous paper [15]. (TIF) [file pone.0092194.s003.tif]

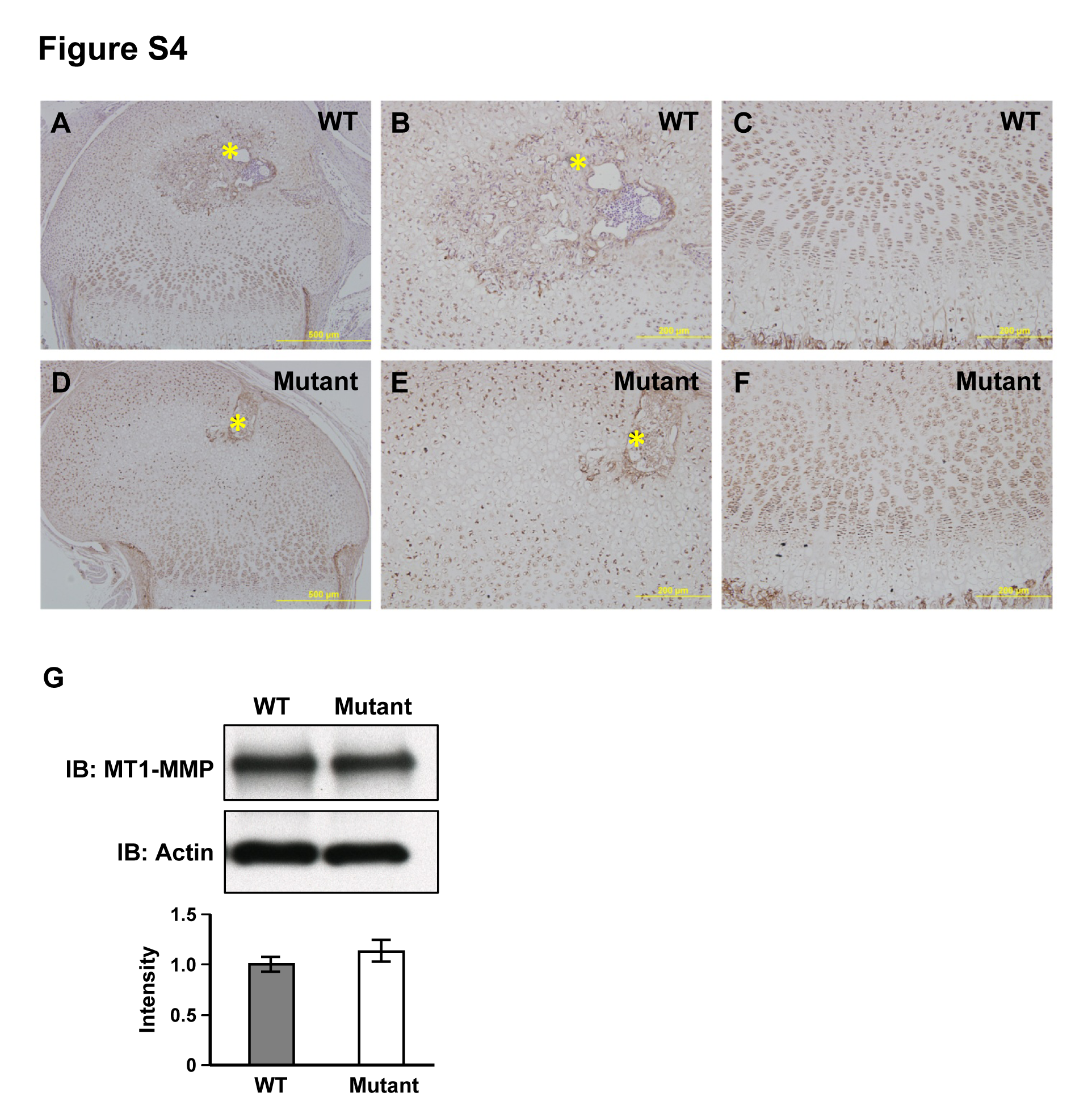

Supplement: Figure S4 — Comparable MT1-MMP expression in WT and mutant epiphysis and primary chondrocytes. MT1-MMP IHC of P10 (A-C) WT and (D-F) mutant distal femoral epiphyseal sections. High power view of (B) WT and (E) mutant mice MT1-MMP expression in chondrocytes around cartilage canal. MT1-MMP expression in (C) WT and (F) mutant growth plate chondrocytes. Yellow star indicted the canal that will contribute to future SOC formation. (G) Expression of MT1-MMP in P14 epiphysis tissue by WB and quantitative results from three WT and mutant littermate pairs (below). (TIF) [file pone.0092194.s004.tif]

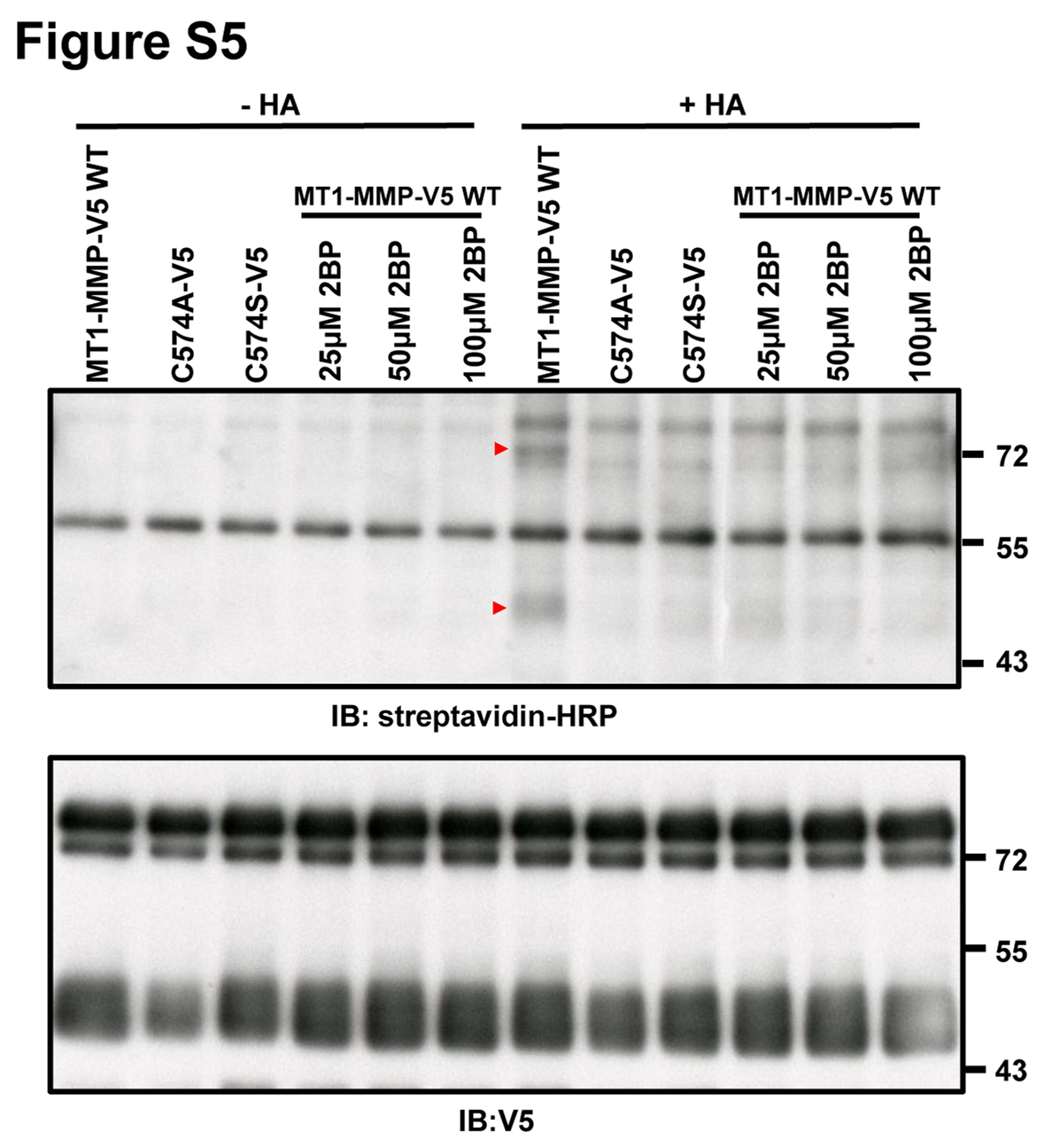

Supplement: Figure S5 — Abolishment of palmitoylation of mutant MT1-MMP (C574A, C574S) and 25 μM, 50 μM, 100 μM 2-Bromopalmitate treatment. Palmitoylation level was examined by acyl-biotin exchange. MT1-MMP-V5 WT or C574A or C574S were overexpressed in HEK293 cells. Palmitate was switched to biotin and detected by streptavidin-HRP. Purified MT1-MMP was analyzed by V5 antibody. Red arrows indicate the palmitoylation signals of WT MT1-MMP which was not detected in C574 mutation constructs and 2-BP treatment groups. The 2-BP concentration of 25 μM was used in further experiment. HA: hydroxylamine; 2BP: 2-bromopalmitate. (TIF) [file pone.0092194.s005.tif]

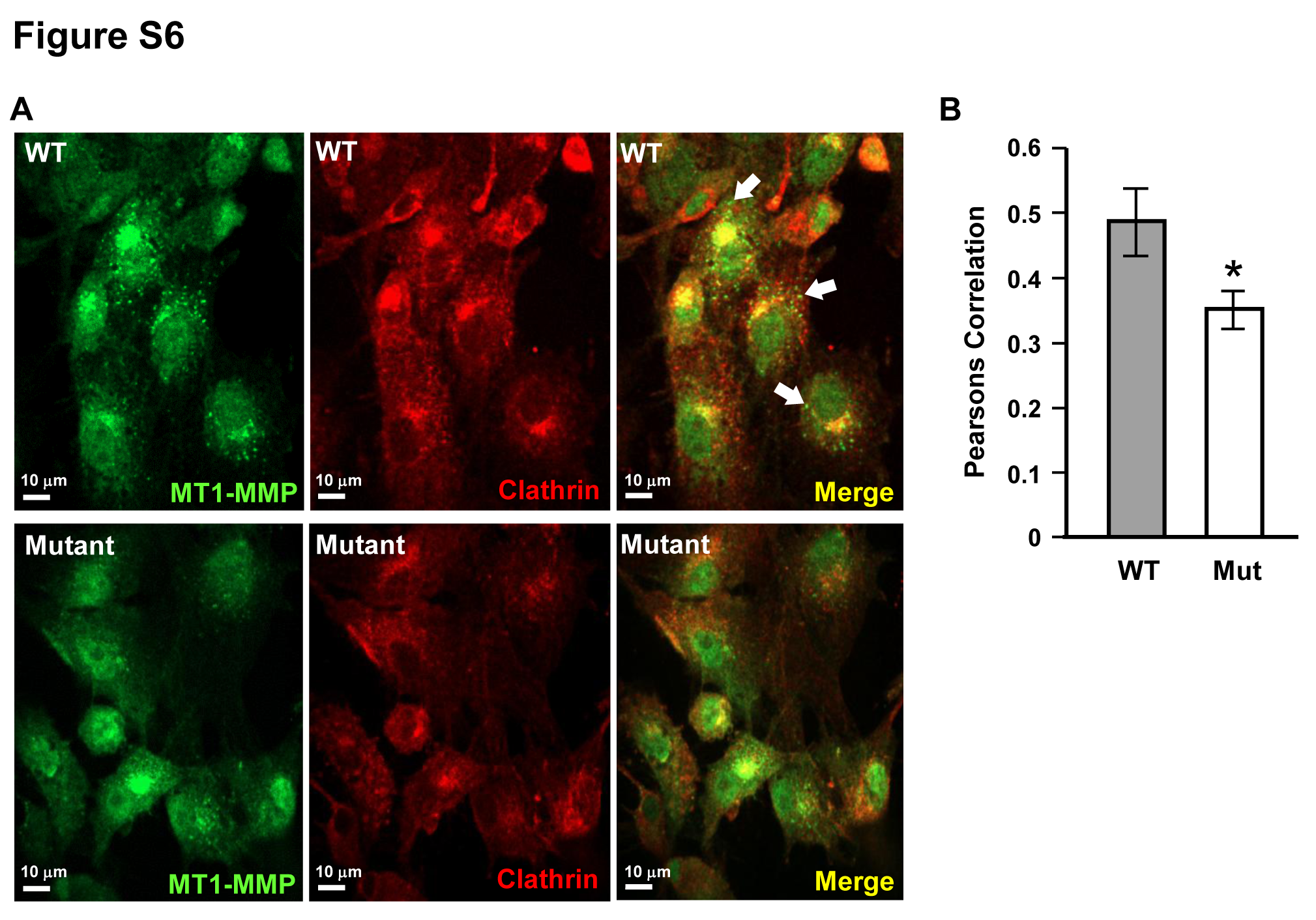

Supplement: Figure S6 — Clathrin-mediated MT1-MMP trafficking in WT and Zdhhc13 mutant primary osteoblast. (A) Localization patterns of MT1-MMP in primary osteoblast (OB) from P14 WT and mutant femur. MT1-MMP staining (green), Clathrin staining (red), and merge of MT1-MMP and Clathrin (yellow shows the colocalization of two proteins) in WT (upper panel) and mutant (lower panel) primary OB. White arrows indicated non-clathrin colocalized speckles. (B) Colocalization MT1-MMP and clathrin quantitative data. *P-value <0.05, t-test. (TIF) [file pone.0092194.s006.tif]
