# Supplementary material for: Palmitoyl Acyltransferase, Zdhhc13, Facilitates Bone Mass Acquisition by Regulating Postnatal Epiphyseal Development and Endochondral Ossification: A Mouse Model
Source: PLoS One. 2014 Mar 17;9(3):e92194. doi: 10.1371/journal.pone.0092194 (PMC3956893; doi:10.1371/journal.pone.0092194)
Supplement: Table S1 — Quantitative microCT results of WT and mutant femur trabecular bone and bone mineral density (BMD) at P10 and P14. 3 WT and 3 mutants were analyzed at each time point. Data was presented as average ± standard deviation. Statistical significance was determined by two-tailed Student's t-test. A P-value <0.05 was considered statistically significant (*P <0.05, **P <0.01). BV/TV: bone volume/ tissue volume; Tb.Th: trabecular bone thickness; Tb.Sp: trabecular separation; Tb.N: trabecular bone number; SMI: structure model index. (DOCX) [file pone.0092194.s007.docx]

**Supplementary Table**

**Table S1.** **Quantitative microCT results of WT and mutant femur trabecular bone and bone mineral density (BMD) at P10 and P14.**

3 WT and 3 mutants were analyzed at each time point. Data was presented as average ± standard deviation. Statistical significance was determined by two-tailed Student's t-test. A P-value <0.05 was considered statistically significant (**P* <0.05, ***P* <0.01). BV/TV: bone volume/ tissue volume; Tb.Th: trabecular bone thickness; Tb.Sp: trabecular separation; Tb.N: trabecular bone number; SMI: structure model index.

| **Age** | **P0** | | **P14** | |
| --- | --- | --- | --- | --- |
| **Genotype** | **WT** | **Mutant** | **WT** | **Mutant** |
| **BV/TV (%)** | 1.28±1.37 | 1.25±1.44 | 6.63±0.93 | 3.42±0.65* |
| **Tb.Th (mm)** | 0.04±0.006 | 0.04±0.007 | 0.06±0.002 | 0.05±0.002** |
| **Tb.Sp (mm)** | 0.28±0.08 | 0.29±0.06 | 0.59±0.004 | 0.64±0.02* |
| **Tb.N (1/mm)** | 0.05±0.02 | 0.06±0.015 | 1.42±0.19 | 0.68±0.12** |
| **SMI** | 2.88±0.13 | 2.67±0.20 | 2.12±0.03 | 2.44±0.05** |
| **BMD (g/cm^3^)** | 0.39±0.06 | 0.38±0.04 | 0.45±0.013 | 0.41±0.008* |
